# Vaccination intention among healthcare workers during the first wave of the coronavirus disease 2019 pandemic in relation to knowledge: a cross-sectional study in Croatia, Slovenia, Serbia, and Poland

**DOI:** 10.3325/cmj.2022.63.79

**Published:** 2022-02

**Authors:** Nevenka Kregar Velikonja, Vislava Globevnik Velikonja, Ivan Verdenik, Ivan Jurišić, Sanja Stanisavljević, Beata Dobrowolska, Karmen Erjavec

**Affiliations:** 1 Faculty of Health Sciences, University of Novo Mesto, Novo Mesto, Slovenia; 2 Division for Obstetrics and Gynaecology, University Medical Centre Ljubljana, Ljubljana, Slovenia; 3 General Hospital Bjelovar, Bjelovar, Croatia; 4 The College of Health Sciences, Academy of Applied Studies Belgrade, Belgrade, Serbia; 5 Department of Management in Nursing, Faculty of Health Sciences, Medical University of Lublin, Lublin, Poland

## Abstract

**Aim:**

To analyze SARS-CoV-2 vaccination intention and acceptance in relation to the knowledge about coronavirus disease 2019 (COVID-19) among healthcare workers (HCWs) in Croatia, Slovenia, Serbia, and Poland.

**Methods:**

In spring 2020, an online survey was distributed among HCWs by using snowball sampling. The questionnaire was fully completed by 623 respondents: 304 from Croatia, 86 from Slovenia, 90 from Serbia, and 143 from Poland. The survey collected data on demographic characteristics (age, gender, education), vaccination acceptance, and knowledge about COVID-19.

**Results:**

A total of 31% of respondents declared their intention to be vaccinated when a vaccine against COVID-19 is available, and 45% were undecided. Vaccination intention was associated with age, educational level, and knowledge about the pandemic, and differed significantly among the countries. Younger HCWs (18-25 years) and those with higher education more frequently expressed vaccination acceptance. Vaccination acceptance score was not associated with gender.

**Conclusions:**

HCWs with higher knowledge were more likely to express vaccination intention. Improving the knowledge about COVID-19 and increasing HCWs' education might also increase vaccination acceptance among HCWs, and consequently in the general population.

When in early March 2020, the SARS-CoV-2 virus began to spread in Europe, the governments of Croatia, Slovenia, Serbia, and Poland relatively quickly imposed containment measures, including a closure of kindergartens and schools, and a ban on public life. The measures seemed to be successful, as in the first wave of the pandemic these countries had lower COVID-19 infection and death rates than some Western European countries such as Italy, France, Spain, and the United Kingdom (1). It soon became clear that, in addition to prevention, diagnosis, and treatment, the pandemic can be limited globally only by the introduction of vaccines against COVID-19 (2). The success of a vaccination program depends on the uptake rates in the population, especially among health care workers (HCWs) (3). Better knowledge about the disease and higher perceived severity of COVID-19 have been shown to increase vaccine acceptance (4).

HCWs play an important role as health educators and can help in disease control by disseminating accurate information in communities. According to the theory of knowledge, attitude, and practice, successful disease control requires good knowledge of the disease (5). HCWs' lack of such knowledge can delay treatment and lead to rapid spread of infection (6,7). Indeed, HCWs were shown to have inadequate knowledge about COVID-19 (8).

HCWs have a higher risk of becoming infected with COVID-19 than the general population (3,9,10) and are potential transmitters of the virus in the clinical setting. However, they can also help the lay population understand and accept vaccination. In Southeast Asia, HCWs had higher acceptance of COVID-19 vaccination than the general population, due to a higher perceived risk of COVID-19 infection (11). Chinese HCWs had higher willingness to receive future vaccination compared with lay population (12). Because HCWs are the most important sources of information and the strongest authority when it comes to vaccination decisions (13-15), their opinions and vaccination intentions should be assessed, and the relation between key sociodemographic factors and vaccination intentions should be investigated.

Due to the importance of HCWs' vaccination uptake during the COVID-19 pandemic in Central and Eastern Europe, this study analyzed HCWs' vaccination acceptance in Croatia, Slovenia, Serbia, and Poland in relation to their knowledge about COVID-19. Based on the findings of previous research on influenza vaccination uptake (17), we hypothesized that the countries would significantly differ in COVID-19 vaccination intention and acceptance among HCWs and that vaccination acceptance would be influenced by gender, education, knowledge, and attitudes.

## PARTICIPANTS AND METHODS

This cross-sectional, web-based study was conducted among HCWs during the first wave of the pandemic (April-May 2020) in four Central and Eastern European countries.

### Participants

An online survey was distributed among HCWs by using snowball sampling, a method used in similar previous studies (6,16,17,18). Project members contacted their professional contacts from different health care institutions on primary, secondary, or tertiary level with a request to share the survey further. Respondents were asked to complete a self-administered, structured electronic questionnaire. The time frame when the survey was active in the participating countries is shown in Table 1. The questionnaire was fully completed by 623 respondents who identified themselves as HCWs: 304 from Croatia, 86 from Slovenia, 90 from Serbia, and 143 from Poland. There were 91% female respondents. The average age was 37.6 years. A total of 37% of the respondents had a secondary school degree, 36% had a graduate degree (corresponding to the first Bologna cycle), and 27% had a postgraduate degree (corresponding to the second Bologna cycle or higher).

**Table 1:** Time frame of survey activity and demographic characteristics of respondents from the participating countries

			No. (%) of respondents			
	Period of survey	No. of healthcare workers	female	secondary school	graduate	postgra duate
Croatia	May 6-May 25, 2020	304	277 (91)	140 (46)	109 (36)	55 (18)
Slovenia	April 13-April 8, 2020	86	75 (87)	57 (67)	15 (17)	14 (16)
Serbia	April 12-May 7, 2020	90	83 (92)	32 (35)	47 (52)	12 (13)
Poland	April 7-May 7, 2020	143	129 (90)	4 (3)	54 (38)	85 (59)
Total	April 7-May 25, 2020	623	567 (91)	231 (37)	224 (36)	168 (27)

### Research instrument

The questionnaire collected data on demographic characteristics (age, gender, level of education), vaccination intention, vaccination acceptance, and knowledge about COVID-19.

Eight statements were used to assess the attitudes towards COVID-19 (Table 2), with response options of "yes", "no," or "I do not know." The vaccination acceptance score was calculated as the sum of the scores for each individual response (response reflecting acceptance of vaccination: +1, do not know: 0, disagreeing with vaccination: -1; range of values: -8 to +8, Cronbach’s alpha = 0.78).

**Table 2:** Vaccination acceptance and attitude towards coronavirus disease 2019 (COVID-19) vaccine by country (n = 628)

	No. (%) of respondents			Chi-square test for testing differences among countries	Correlation with the statement "I will definitely get vaccinated"	Correlation with the statement "I am categorically against the use of vaccines"
	yes	no	don't know	p	Spearman rho	Spearman rho
**I will definitely be vaccinated.**						
Croatia (n=304)	42 (13.8)^‡^	107 (35.2)	155 (51.0)	0.001		
Slovenia (n=87)	38 (43.7)	14 (16.1)	35 (40.2)			
Serbia (n=94)	39 (41.5)	10 (10.6)	45 (47.9)			
Poland (n=143)	76 (53.1)	17 (11.9)	50 (35.0)			
Total (n=628)	195 (31.1)	148 (23.6)	285 (45.4)			-0.327^*^
**Vaccination should be mandatory for all.**						
Croatia (n=304)	44 (14.5)	146 (48.0)	114 (37.5)	0.001		
Slovenia (n=87)	26 (29.9)	33 (37.9)	28 (32.2)			
Serbia (n=94)	39 (41.5)	20 (21.3)	35 (37.2)			
Poland (n=143)	66 (46.2)	29 (20.3)	48 (33.6)			
Total (n=628)	175 (27.9)	228 (36.3)	225 (35.8)		0.371^*^	-0.200^*^
**Vaccination should be recommended for children.**						
Croatia (n=304)	61 (20.1)	93 (30.6)	150 (49.3)	0.001		
Slovenia (n=86)	31 (36.0)	15 (17.4)	40 (46.5)			
Serbia (n=94)	23 (24.5)	10 (10.6)	61 (64.9)			
Poland (n=143)	61 (42.7)	23 (16.1)	59 (41.3)			
Total (n=627)	176 (28.1)	141 (22.5)	310 (49.4)		0.219^*^	-0.181^*^
**Vaccination should be recommended for people over 65.**						
Croatia (n=304)	249 (81.9)	15 (4.9)	40 (13.2)	0.879		
Slovenia (n=87)	73 (83.9)	3 (3.4)	11 (12.6)			
Serbia (n=94)	79 (84.0)	2 (2.1)	13 (13.8)			
Poland (n=143)	118 (82.5)	4 (2.8)	21 (14.7)			
Total (n=628)	519 (82.6)	24 (3.8)	85 (13.5)		0.171^*^	-0.130^*^
**Vaccination should be recommended for people with chronic diseases.**						
Croatia (n=304)	257 (84.5)	15 (4.9)	32 (10.5)	0.114		
Slovenia (n=87)	76 (87.4)	3 (3.4)	8 (9.2)			
Serbia (n=94)	78 (83.0)	1 (1.1)	15 (16.0)			
Poland (n=143)	117 (81.8)	2 (1.4)	24 (16.8)			
Total (n=628)	528 (84.1)	21 (3.3)	79 (12.6)		0.569^*^	-0.223^*^
**I believe the vaccine will be effective.**						
Croatia (n=304)	44 (14.5)	45 (14.8)	215 (70.7)	0.001		
Slovenia (n=87)	28 (32.2)	10 (11.5)	49 (56.3)			
Serbia (n=94)	29 (30.9)	13 (13.8)	52 (55.3)			
Poland (n=142)	62 (43.7)	14 (9.9)	66 (46.5)			
Total (n=627)	163 (26.0)	82 (13.1)	382 (60.9)		0.457^*^	-0.335^*^
**I believe the vaccine will be safe.**						
Croatia (n=304)	46 (15.1)	62 (20.4)	196 (64.5)	0.001		
Slovenia (n=87)	27 (31.0)	17 (19.5)	43 (49.4)			
Serbia (n=94)	33 (35.1)	11 (11.7)	50 (53.2)			
Poland (n=142)	67 (47.2)	11 (7.7)	64 (45.1)			
Total (n=627)	173 (27.6)	101 (16.1)	353 (56.3)		0.475^*^	-0.396^*^
I am categorically against the use of vaccines.						
Croatia (n=304)	37 (12.2)	177 (58.2)	90 (29.6)	0.001		
Slovenia (n=87)	6 (6.9)	75 (86.2)	6 (6.9)			
Serbia (n=94)	8 (8.5)	67 (71.3)	19 (20.2)			
Poland (n=143)	2 (1.4)	134 (93.7)	7 (4.9)			
Total (n=628)	53 (8.4)	453 (72.1)	122 (19.4)		-0.327^*^	

*Correlation is significant at the 0.01 level (2-tailed).

†Correlation is significant at the 0.05 level (2-tailed).

‡Bold statements indicate the answer reflecting vaccination acceptance or positive attitude towards vaccination.

Eleven statements were used to assess the knowledge about COVID-19, epidemiological situation, and appropriate preventive measures (Table 3), with the response options "I agree," "I disagree," or "I do not know." The set of statements was based on the current relevant knowledge about SARS-CoV-2 and epidemiological data (3-8). The knowledge score was calculated as the sum of the scores for each individual response (correct answer: +1, do not know: 0, wrong answer: -1; range of values: -11 to 11). We asked about different facts that were communicated differently to the public during the first wave of the pandemic. Therefore, the internal consistency of this construct was not expected.

**Table 3:** Statements for assessment of knowledge about coronavirus disease 2019 (COVID-19)

		I agree*	I don't agree*	N (%) of correct answers				
				Total (n=627)	Croatia (n=304)	Slovenia (n=87)	Serbia (n=93)	Poland (n=143)
S1	There is currently a worldwide pandemic of COVID-19.	x		581 (92.5)	271 (89.1)	80 (92.0)	91 (96.8)	139 (97.2)
S2	There is currently an epidemic of COVID-19 disease in my country.	x		542 (86.4)	245 (80.6)	78 (89.7)	90 (96.8)	129 (90.2)
S3	Healthy people do not get infected with COVID-19.		x	565 (90.0)	261 (85.9)	78 (89.7)	90 (95.7)	136 (95.1)
S4	Healthy people cannot be carriers of the new coronavirus.		x	544 (86.8)	242 (79.6)	82 (94.3)	88 (94.6)	132 (92.3)
S5	There are effective antiviral medicines for treatment of COVID-19.		x	443 (70.5)	198 (65.1)	77 (88.5)	60 (63.8)	108 (75.5)
S6	SARS-CoV-2 infection is thought to be transmitted to humans through animals.	x		187 (29.8)	58 (19.1)	32 (36.8)	27 (29.0)	70 (49.0)
S7	Mortality is higher with COVID-19 than with influenza.	x		297 (47.3)	125 (41.1)	38 (43.7)	56 (59.6)	78 (54.5)
S8	Coronavirus belongs to RNA viruses.	x		319 (50.9)	134 (44.1)	35 (40.2)	60 (64.5)	90 (62.9)
S9	Coronavirus can be destroyed by 60% alcohol disinfectant.	x		354 (56.4)	159 (52.3)	51 (58.6)	28 (29.8)	116 (81.1)
S10	Coronavirus can be destroyed by freezing.		x	321 (51.2)	136 (44.7)	52 (60.5)	51 (54.3)	82 (57.3)
S11	In addition to coronavirus and influenza virus, other viruses can also cause respiratory diseases.	x		602 (95.9)	292 (96.1)	80 (92.0)	90 (95.7)	140 (97.9)

*x – correct answer.

The study protocol was approved by the Ethics Committee of the Faculty of Health Sciences, University of Novo Mesto (FZV-98/2020).

### Statistical analysis

Descriptive analysis was presented as frequencies and proportions. The chi-square test and ANOVA were used to assess the differences between the countries in categorical and numerical variables, respectively. The correlations between different vaccination-related items were assessed with the Spearman correlation coefficient. Multivariate logistic regression analysis was used to identify factors influencing vaccine acceptance, vaccination intention, and vaccine refusal. A P value < 0.05 was considered significant. The data were analyzed with SPSS, version 25.0 (IBM Corp, Armonk, NY, USA).

## RESULTS

### Vaccination intention and vaccination acceptance

Vaccination intention was assessed with the statement "I will definitely be vaccinated." About one-third of all respondents (31.1%) indicated that they intended to be vaccinated, and 45.4% were undecided (Table 2). The countries (P < 0.001) significantly differed regarding vaccination intention: respondents from Croatia least frequently expressed vaccination intention (13.8%), while those from Poland (53.1%) most frequently expressed vaccination intention. Croatian respondents also least frequently stated that vaccination should be mandatory for all, that it should be recommended for children, that it would be safe, and that it would be effective. Respondents from Poland most frequently expressed these opinions. The percentage of respondents categorically refusing vaccination was highest in Croatia (12.2%) and lowest in Poland (1.4%). The countries did not significantly differ in the percentage of respondents (over 80% in all countries) who agreed with the recommendation to vaccinate people with chronic diseases and those over 65 years (Table 2).

Vaccination intention was significantly correlated with all other statements; the highest correlation was found with the statements "Vaccination should be recommended for people with chronic diseases;" "I believe the vaccine will be effective," and "I believe the vaccine will be safe." The statement "I am categorically against the use of vaccines" was significantly negatively correlated with all the other statements for the evaluation of vaccination acceptance (Table 2). The highest vaccination acceptance score was observed in Poland, and the lowest in Croatia, significantly lower than in other countries (Bonferroni *post-hoc* test, Table 4).

**Table 4:** Overall assessment of vaccination acceptance and knowledge scores in participating countries

		Vaccination acceptance score			Knowledge score		
	N	mean	standard deviation	ANOVA (P)	mean	standard deviation	ANOVA (P)
Croatia	304	1.32	2.96	0.001	5.13	2.70	0.001
Slovenia	86	3.11	3.29		6.48	2.77	
Serbia	90	3.32	3.11		6.19	1.92	
Poland	143	4.17	2.89		7.23	2.27	
Total	629	2.52	3.24		5.95	2.66	

### Knowledge about the COVID-19 pandemic

A high percentage of respondents were informed about the pandemic and the general characteristics of viral infectivity (statements S1, S2, S3, S4, S11); they were less informed about biological facts (S6, S8) and disinfection procedures (S9, S10) (Table 3). Seventy percent knew that there was no effective antiviral drug on the market (S5), but less than half thought that the mortality from COVID-19 was higher than that from influenza (S7) (Table 3). Polish respondents had the best knowledge about the COVID-19 pandemic, and Croatian respondents had the poorest knowledge, significantly poorer than in other countries (Bonferroni *post-hoc* test, Table 4).

### Association of vaccination acceptance and knowledge score with demographic data

The 18-25-year-old respondents had a significantly higher vaccination acceptance than older age groups (Bonferroni *post-hoc* test, P = 0.023). Men and women did not significantly differ (P = 0.288). Respondents with secondary school education had a significantly lower vaccination acceptance than respondents with graduate (P = 0.019) and postgraduate education (P = 0.020, Bonferroni *post-hoc* test). The knowledge score significantly positively correlated with the vaccine acceptance score (r = 0.354; P < 0.001) (Table 5).

**Table 5:** Association between vaccination acceptance and knowledge scores and demographic characteristics

			Vaccination acceptance score		ANOVA	Knowledge score		ANOVA
		N	mean	standard deviation	P	mean	standard deviation	P
Age	18-25	122	3.57	3.352	<0.001	5.92	2.323	0.632
	26-30	98	2.01	3.248		6.00	2.685	
	31-40	162	1.85	3.298		5.73	2.802	
	>40	239	2.62	2.999		6.08	2.750	
	total	621	2.51	3.241		5.94	2.672	
Gender	male	58	2.95	3.220	0.316	6.16	2.858	0.526
	female	566	2.47	3.244		5.92	2.647	
	total	624	2.52	3.242		5.94	2.666	
Education level	secondary	233	1.99	3.210	0.006	5.53	2.626	<0.001
	graduate	227	2.80	3.242		5.83	2.616	
	postgraduate	166	2.89	3.195		6.70	2.636	
	total	626	2.52	3.239		5.95	2.663	

A multivariate regression analysis carried out to determine the parameters influencing vaccination acceptance (reference: vaccination acceptance score > 0) including all the factors (gender, education, knowledge, country, age) showed that only the knowledge score and the country of origin predicted a positive attitude towards vaccination (Table 6). Although vaccination acceptance differed significantly according to educational level, the education level was not a predictor for COVID-19 vaccination acceptance.

**Table 6:** Multivariate logistic regression analysis of factors influencing vaccine acceptance, vaccination intention, and vaccine refusal

		Positive vaccination acceptance score*		Vaccination intention^†^		Categorical vaccine refusal^‡^	
		P	odds ratio (95% confidence interval)	P	odds ratio (95% confidence interval)	P	odds ratio (95% confidence interval)
Education	postgraduate (ref)	0.971		0.118		0.701	
	secondary	0.868	1.050 (0.592-1.862)	0.239	0.605 (0.262-1.396)	0.587	0.784 (0.326-1.885)
	graduate	0.809	1.072 (0.612-1.878)	0.566	1.258 (0.575-2.750)	0.400	0.685 (0.284-1.653)
Knowledge score		0.000	1.330 (1.193-1.483)	0.000	1.695 (1.426-2.028)	0.000	0.685 (0.581-0.807)
Age	> 40 (ref)	0.564		0.003		0.826	
	18-25	0.832	1.068 (0.581-1.962)	0.135	1.924 (0.816-4.535)	0.823	0.896 (0.341-2.354)
	26-30	0.892	.960 (0.532-1.731)	0.271	0.606 (0.249-1.477)	0.466	1.391 (0.573-3.374)
	31-40	0.228	0.744 (0.459-1.204)	0.018	0.425 (0.209-0.866)	0.635	1.203 (0.561-2.581)
Country	Croatia (ref)	0.001		0.000		0.027	
	Slovenia	0.098	1.669 (0.911-3.058)	0.000	5.823 (2.457-13.801)	0.168	0.513 (0.199 -1.326)
	Serbia	0.007	2.489 (1.283-4.831)	0.000	5.462 (2.262-13.193)	0.947	1.030 (0.428-2.481)
	Poland	0.001	3.109 (1.585-6.101)	0.000	4.520 (2.008-10.177)	0.006	0.115 (0.025-0.533

*Vaccination acceptance score 1 or more.

†Statement "I will definitely get vaccinated."

‡Statement "I am categorically against vaccines."

**Figure 1:**
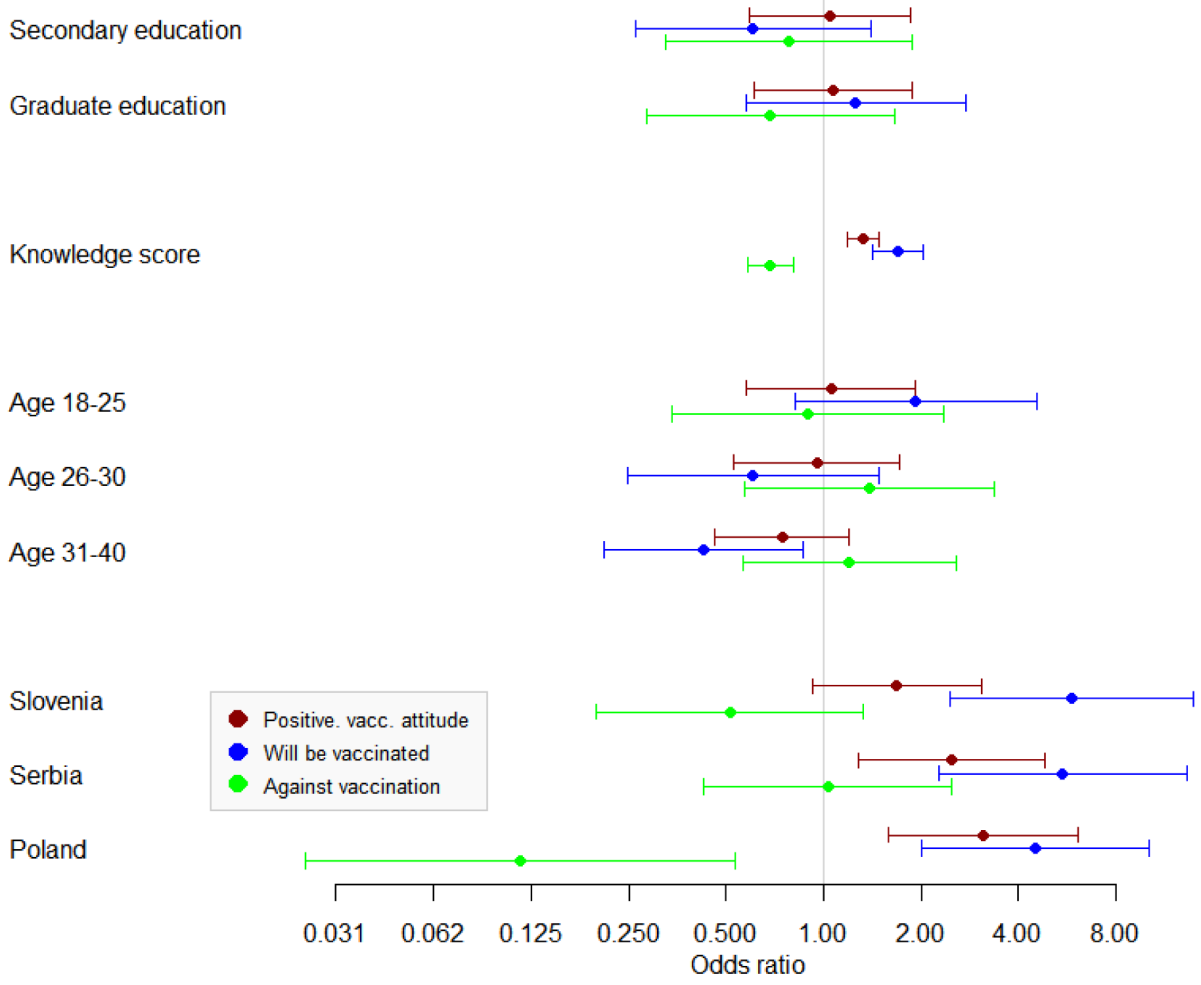
Logistic regression analysis of factors influencing vaccination acceptance, vaccination intention, and vaccination refusal (reference categories: education: postgraduate, age: 41 and more, country: Croatia; in knowledge score odds ratio is given for 1 point increase).

The knowledge score and the country of origin were also predictors for vaccination intention (agreement with the statement "I will definitely be vaccinated") and for categorical vaccine refusal (agreement with the statement "I am categorically against vaccines"). An additional predictor for vaccination intention was age (Table 6, Figure 1).

## DISCUSSION

In this study, 31% of respondents declared their intention to be definitely vaccinated once a vaccine against COVID-19 is available, whereas 45% were undecided. The vaccination acceptance score was associated with age, educational level, and the knowledge about the pandemic, and it differed significantly among the countries. HCWs aged 18-25 years and those with higher education expressed a higher vaccination acceptance. An association between vaccination acceptance and gender was not confirmed. The knowledge score significantly positively correlated with the vaccination acceptance score, indicating that HCWs with higher knowledge levels had a more positive attitude toward vaccination. This finding is consistent with the findings of a COVID-19 related study of the general population in the United States (4), and previous research on HCWs (19,20). HCWs' adequate knowledge about the disease is important as HCWs are still the most trusted advisors and influencers when it comes to vaccination decisions in the face of increasing vaccination hesitancy (21). We confirmed the previous findings that lower education levels positively correlated with negative attitudes towards vaccination (22).

The sum of the rates of the respondents who intended to be vaccinated and of the undecided respondents obtained in this study (76.5%) is similar to the rates observed in other recent studies in Europe and the United Kingdom (23,24). Namely, 73.4% of the general population in 7 European countries (23), and 76.9% in the UK (24) was willing to be vaccinated against COVID-19. In a Canadian study, more than two-thirds of crowdsourcing participants were very willing to get vaccinated (25). In Far East countries, the rates of definite vaccination intention were higher: 48.2% among general population in Malaysia (26) and 40% among nurses in Hong Kong (27). However, these data are not directly comparable due to differences in survey designs. Nonetheless, HCWs usually have more positive attitudes towards vaccination than the general population (20,28), but vaccination rates among HCWs are often low (29).

Vaccination intention and acceptance depend on the public trust in the safety and efficacy of vaccines, but also on the trust in the health care system, HCWs, and the broader vaccine research community (30,31). Concerns about the vaccine safety have decreased the vaccine uptake (32,33). In this survey, only about a quarter of respondents believed that the vaccine would be effective and safe.

The willingness to be vaccinated during the first wave of the COVID-19 pandemic varied widely among the participating countries: the highest vaccine acceptance was reported by Polish, and the lowest by Croatian respondents. Influenza vaccination acceptance among HCWs was also affected by the country of origin (34). Varying levels of vaccination acceptance have also been reported in the general population of different European countries (23).

Trust in the safety and efficiency of the vaccine is one of the major factors influencing vaccination intention (12,22,24,31,35,36). Differences in the willingness to be vaccinated could be explained by different cultural and social parameters. Trust in health and government authorities and institutions involved in vaccination was significantly associated with vaccination intention (25,35). Distrust in formal institutions (ie, health scientists, health and pharmaceutical industries, government) is associated with high levels of neophobia (36), which suggests an interaction effect of trust and neophobia on the willingness and acceptance of anything new, including a new vaccine (37).

Increasing the knowledge about the benefits and risks of vaccination should be one of the major solutions for tackling vaccination hesitancy (17). The knowledge level is associated with vaccination intention (22). The highest knowledge level and the highest vaccination acceptance rate in Poland and the lowest in Croatia can be partly explained by significantly higher education level of Polish respondents than that of respondents from other participating countries, as a higher education level was associated with a higher vaccination acceptance. However, previous research also confirms different vaccination attitudes in the participating countries (14,15,19,33,38).

In Croatia, the number of people vaccinated against influenza has been steadily decreasing since 2010 as anti-vaccination attitudes were strengthening (38). Vaccination hesitancy was observed among HCWs in Croatia, France, Greece, and Romania (34). The main concern in these countries was the fear of side effects. In addition, while health workers trusted health authorities, they distrusted pharmaceutical companies due to perceived financial interests and lack of communication about side effects (33). On the other hand, 86% of Polish physicians strongly supported vaccination; 62% received seasonal influenza vaccine every year (19). Interestingly, 70.1% of Polish lay people also supported vaccination (15).

The highest share of HCWs who categorically rejected the possibility of vaccination was found in Croatia (12.2%). In contrast, only 2.2% of HCWs in Croatia were against vaccination in children (20).

In our study, regression analysis showed the knowledge score and the country of origin to be the predictors of vaccination acceptance, vaccination intention, and categorical vaccine refusal. An additional predictive factor of vaccination intention was age. Therefore, vaccination intention could be increased by launching programs for increasing HCWs’ knowledge about COVID-19 and SARS-CoV-2. As vaccination intention appears to be highly associated with the belief in the safety and efficacy of the vaccine, HCWs should be appropriately informed about these issues. Increasing the HCWs' knowledge might further increase their capacity and confidence to adequately answer the patients’ questions (14-16,22).

Several other factors were reported to influence vaccination acceptance. Influenza vaccination hesitancy among HCWs was associated with low-risk perception of the disease (32). The willingness to be vaccinated was increased by triggering altruistic motives (40). The most effective strategy was to explain to HCWs that by getting vaccinated they would reduce the danger for individuals who cannot be vaccinated (39). The mentioned factors should also be considered in future research on vaccine acceptance and hesitancy.

Our study was limited by the snowball sampling procedure, which is a non-random sampling method. However, in all participating countries the respondents were enrolled in the same manner. Additionally, at the time of the survey the four countries did not go through the same stage of the epidemic, since in Croatia the survey was performed with a slight delay. Data on the type of health care institution where respondents worked were not collected, although this factor could also have influenced the respondents' attitudes. The variation in this variable could have led to differences between countries. The survey was conducted during the late phase of the first wave of the COVID-19 pandemic. As no vaccination was available at this time, respondents’ assumptions on vaccine efficacy and safety were only hypothetical. Therefore, both vaccination intention and attitudes towards the vaccine might have changed in the later phases of the pandemic when more scientific knowledge on the vaccine was available.

In conclusion, HCWs with higher levels of knowledge were more likely to be vaccinated. The activities to improve the knowledge about the new disease and to promote higher education of HCWs might positively influence the vaccination acceptance rate among HCWs, and consequently in the general population.
